# Using and understanding cross-validation strategies. Perspectives on Saeb et al.

**DOI:** 10.1093/gigascience/gix020

**Published:** 2017-03-17

**Authors:** Max A Little, Gael Varoquaux, Sohrab Saeb, Luca Lonini, Arun Jayaraman, David C Mohr, Konrad P Kording

**Affiliations:** 1 Department of Mathematics, Astun University, Aston Triangle, B4 7ET, Birmingham, UK; 2 Parietal, INRIA, NeuroSpin, bat 145 CEA Saclay, 91191, Gif sur Yvette, France; 3 Department of Preventive Medicine, Northwestern University, 750 N Lake Shore Dr, 60611, Chicago, USA; 4 Rehabilitation Institute of Chicago, 345 E Superior, 60611, Chicago, USA

**Keywords:** machine learning, clinical applications, cross-validation

## Abstract

This three-part review takes a detailed look at the complexities of cross-validation, fostered by the peer review of Saeb et al.’s paper entitled “The need to approximate the use-case in clinical machine learning.” It contains perspectives by reviewers and by the original authors that touch upon cross-validation: the suitability of different strategies and their interpretation.

This review is organized in three sections, each presenting a different view on the suitability of different cross-validation strategies: one by M.A. Little, one by G. Varoquaux (who both also reviewed the original paper), and one by Saeb et al.

## Perspective by M. A. Little: an important problem that subject-wise cross-validation does not fix

In their important article, Saeb et al. 2017 [1] propose, on the basis of empirical evidence and a simulation study, that one should use leave-subject-out (or “subject-wise”) cross-validation (CV) rather than basic CV (what we are calling “record-wise CV”) in clinical diagnostic application settings of machine learning predictors, where there are multiple observations from each individual and small numbers of individuals. The reason is that complex predictors can pick up a confounding relationship between identity and diagnostic status and so produce unrealistically high prediction accuracy, and this is not correctly reported by record-wise CV, but it is for subject-wise CV. From Saeb et al.’s article [1], I interpret the following claims: (i) subject-wise CV mimics the “use-case”: in usage, predictions will always be made on new subjects for which we have no observations, so observations from individuals in the training set must not appear in the test set, (ii) subject-wise CV will correctly estimate the out-of-sample prediction error under these circumstances, and (iii) record-wise CV creates dependence between training and test sets due to shared identities across train/test sets, so it will produce biased estimates (e.g., prediction errors are underestimated).

**Figure 1:**
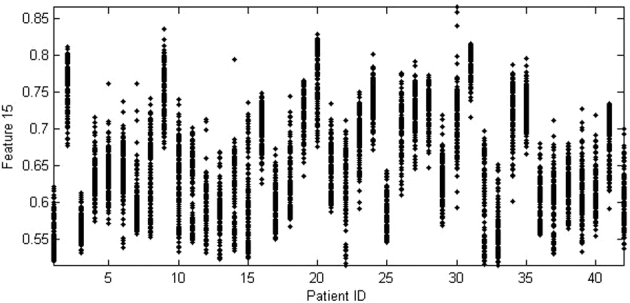
Features from clinical data sets of the kind discussed by Saeb et al. are often highly clustered, often not effectively sharing the same distribution across individuals. Using subject-wise CV with this kind of data, the assumption of subject-wise CV that the training and test sets come from the same distribution is effectively violated. Here the leave-one-subject-out train/test distribution mismatch is 100% for all subjects for this feature (two-sample Kolmogorov-Smirnov test at 0.05 significance, Bonferroni corrected). This means that leave-one-subject-out CV cannot provide a consistent out-of-sample estimator for this data. The mean absolute serial (Pearson) correlation within subject for each feature for all subjects is an unimportant 0.06, with no correlations reaching significance (at the 0.05 level, Bonferroni corrected), barring only one feature that is weakly correlated just above significance for only 10% of subjects. This means that observations within subjects are effectively independent, but such dependence is cited as the main motivation for using subject-wise CV. Data from [3]

When I was asked to critically review this paper originally, I could not really grasp the assumptions of their probabilistic arguments, which were not made explicit. I guess if I were quicker I would have gotten it immediately, but fortunately and later during the review process they relieved my stupidity and made it clear that they intend a specific model for the data in which dependency exists between each observation from each subject, but where subjects – and the full dependency between and distributional structure of observations – are drawn independent and identically distributed ( i.i.d.) from the population as a whole. But whether this model is applicable to any specific data set is empirically testable and they do not propose a direct test for this, so below I want to posit a different model where Saeb et al.’s [1] claims (i-iii) above do not hold. This model is grounded in my practical experience with this kind of data and evidence from real data in this discipline (see Fig. 1). This model allows me to test the scope of Saeb et al.’s [1] claims.

### Subject-wise CV is not always a valid substitute for record-wise CV in diagnostic applications

Take a collection of i.i.d. random variables and observations generated by them. Now split these variables up into two groups, according to any uniformly random selection that is independent of the data. The groups are now independent, and each observation is identically distributed to every other observation in each group. This (i.i.d. situation) describes exactly what record-wise CV assumes [2]. So any uniformly random way of splitting the data into, i.e., test and training subsets, which are independent of the data, must leave each observation in each group independent of and identically distributed to any other observation in either group. The consequence is that record-wise CV should not depend upon how we split the data, provided that we split in a way that is independent of the data. Any estimator of any quantity computed on either group of this split must be independent of the way in which this split is performed [1].

Let us now split the data in a way that is conditioned on one of the variables in the data or some other variable upon which the data depends. The split is no longer in general independent of the data. A simple example is splitting on subject identity. This modification of record-wise CV is indeed subject-wise CV, and we ideally want it to inherit the i.i.d. assumption of record-wise CV, for then we can simply borrow the applicable theory wholesale. But we will find that for some kinds of data we can create a split that violates the “identically distributed” assumption of record-wise CV: indeed, this happens where the data is clustered in value by identity, yet where the data is still i.i.d. For this kind of data, observations belonging to one identity will have different distributions than those in other identities: this is a commonly encountered situation in the context of Saeb et al.’s article [1]; indeed, this kind of clustering is sufficient to cause complex predictor identity confounding (see Fig. 1 for a real example). Such a model is known as an i.i.d. ‘mixture model’ in the statistics and machine learning literature [2].

But, does subject-wise CV work for this data? No: we get generally different values of the estimator in the training set than in the test set because they are based on data with different distributions. Yet, the original data is indeed still i.i.d. To get the same estimator in both groups, we must have different samples from the same distribution, rather than different samples from different distributions, across the train/test split. This contradicts claim (ii) above: in the identity-clustered data case (that can cause identity-confounded predictions), subject-wise CV does not produce a consistent estimate of the out-of-sample prediction error. This distributional mismatch is clear in Fig. 1.

### Clustering does not necessarily imply dependence

Staying for now with this simple i.i.d. mixture model, we can ask about the independence of training and test sets, which bears on claim (iii) above. Does the fact that the data is clustered imply that observations are dependent? The answer is of course no because it is an i.i.d. model. While observations within a cluster might take on more similar values to observations in that cluster than to others, each observation is still independent of every other. What about within clusters? Still no, because it is an i.i.d. model. Indeed, no dependence between observations is created just by clustering alone. To be clear: in this model, observations do depend upon their identity variable – that is what we mean by clustering on identity here – but they do not depend upon each other [3].

This means that no matter how we uniformly, randomly, and independently split the data generated by a simple i.i.d. mixture model, the data in each group are still independent of each other, and they are still independent across groups. But this is the record-wise CV splitting method, and the “independent” part is satisfied. Claim (iii) is contradicted: clustering in this model does not by itself necessarily invalidate record-wise CV due to dependence across a split between training and test sets. And this of course entirely obviates the need for subject-wise CV, contradicting claim (i). However, if we use subject-wise CV anyway, we end up violating the “identically distributed” part by splitting on the clustering variable, contradicting claim (ii).

### For a simple model that exhibits the identity- confounding effect, subject-wise doesn’t work

With just the simple i.i.d. mixture model, the data is clustered and can exhibit identity confounding for complex predictors, but the data is still i.i.d., so no dependence is created between training and testing subsets, which eliminates the theoretical justification for using subject-wise CV. However, to avoid the confounding discovered empirically, subject-wise CV is proposed, which splits on the clustering variable. But this leads inevitably to violating the identically distributed assumption, which is required for subject-wise CV to produce consistent estimates of the out-of-sample prediction accuracy.

Saeb et al. [1] of course may not agree with these model assumptions, particularly the lack of within-subject dependence. It may seem “intuitively obvious” that there must be such dependence. But this is an intuition that is testable, and in my experience, real data I have encountered in this context do not exhibit any detectable signs of within-subject dependence between observations (see, for example, Fig. 1, where the serial correlation within features is effectively non-existent) [4].

In my view, if one cannot actually detect any dependence in practice, then Occam’s razor suggests that we go with the simpler model, which explains the data, i.e., the i.i.d. mixture model. One could still insist that no, there must be some kind of dependence only we haven’t looked hard enough for it. Then these hidden dependencies cause effective clustering by value within subject, and if this dependence is strong enough to cause clustering, which in turn causes identity-confounding for complex predictors, then it is likely to be strong enough for subject-wise CV to empirically violate the identically distributed assumption in any finite sample.

Subject-wise CV-as a proposed solution to identity confounding for complex predictors, for some quite simple models that fit the description given by Saeb et al. [1], fit the data, and cause identity confounding-introduces a new problem that undermines its consistency as an estimator of out-of-sample prediction accuracy, either in theory or empirically, or both. Subject-wise CV does not always work, and in some realistic cases it fails where record-wise CV does not, even when the data are clustered on identity. This sets a significant limitation on the applicability of Saeb et al.’s [1] subject-wise CV proposal.

### The real problem is confounded predictions, not CV

CV is such a seductively simple and universal technique that it is easy to overstate what it can really tell us or do for us in practice. As Saeb et al.’s [1] empirical evidence shows quite clearly, there are practical situations in which there is an apparently systematic difference between prediction error estimates obtained by record-wise CV and by subject-wise CV. But if we strip this down, all that is actually shown is indirect evidence from prediction errors. The synthetic simulation model arguments proposed in the article are interesting, but this is just a toy model, carefully constructed to highlight the identity-confounding flaw with complex predictors. The evidence is again all indirect through prediction errors. So there is no direct evidence in the article that the critical assumptions of various forms of CV are being violated in empirical studies found in the literature.

What is not in doubt, as Saeb et al. [1] show, is that special properties of the data (here, clustering in feature space) interact with specific properties of some prediction algorithms to cause the predictor to settle on a confounded relationship between the input features and the output variable. Because this problem does not lie with the CV method itself, we should find the actual source of the confound and fix that first.

Adapting CV to attempt to fix a particular confound that it cannot fix risks causing additional problems, in this particular case, an inadvertent mismatch between train and test distributions for real data. Modern nonlinear predictors are particularly sensitive to such “data drift,” a well-known problem [4]. Because of this mismatch, subject-wise CV causes systematic underfitting in practice in this situation: an alternative explanation for Saeb et al.’s empirical findings that we cannot rule out. It is not hard to see why this underfitting might occur: if a critical part of the pattern that relates features to the output variable is missing in the training data because an entire set of observations has been removed as they just happen to belong to a specific individual, then no predictor can be expected to make accurate predictions.

Leave-one-subject-out CV is not a panacea and suffers from additional problems. It often results in estimators with large variance because of the considerable heterogeneity between individuals in practice and the often small number of individuals available to each study. It is dubious to interpret a prediction error estimate with large spread.

If one truly can identify CV as the problem, then it would be important to choose another CV method, as Saeb et al. [1] suggest. Theoretical CV research has moved on considerably since record-wise CV was first described, and there are now adapted CV techniques available for dealing with many different scenarios where record-wise or subject-wise CV would not work. Indeed, subject-wise has recently been theoretically investigated for the case where jointly observations from each individual are mutually dependent and individuals are i.i.d. examples of this joint distribution [5]. (I would like to thank the authors for making me aware of this one.) And there are adapted methods for dealing with mismatched distributions [6] or serial dependencies between observations where we want to make prognostic predictions (e.g., the time series setting or longitudinal setting; see [2]). I would also want to make readers aware of the related ideas of domain adaptation [7], a very active topic addressing the many issues that arise in deployment situations, where the deployment data differ substantially in distribution and other aspects from the train/test data.

### Mandating subject-wise CV could make things worse

While I find the pleasing symmetry of a simple idea such as ‘the CV method must match/approximate the use-case’ very appealing, of which Saeb et al.’s subject-wise CV proposal is a special case, I don’t believe it is for the best to follow this prescription uncritically. In practice, we usually face multiple known and unknown potential confounds.

Such perplexing confounds seem to have taxed the minds of experts in the field for a long time. They have, in particular, been the subject of substantial investigation in the data mining community, where they are known as “leakages,”(see [8] for many concrete examples and examples where attempts to fix them actually exacerbate the problem). For a hypothetical but entirely plausible example, consider the case where all the healthy subjects in the data set are younger than those with the condition we want to diagnose. In this case, since many aspects of aging can be detected in features, we could have an obvious age-related confound in the predictor, and again, subject-wise CV will not fix this. Worse though, everyone could come to believe the results are technically sound solely because it uses subject-wise CV according to prescription.

It may be that a purely pragmatic solution, where appropriate given the probabilistic dependency and distributional structure of the data, is to try out both record-wise and subject-wise CV on a problem just to see what you find. If an unexpected discrepancy is found, this may indicate that some kind of confound based on identity is occurring. Then one should fix the confound.

In summary, this article contains some important observations and interesting empirical evidence, and we should be grateful to Saeb et al. [1] for their work here, and it opens up an important discussion. However, I do not agree with the uncritical application of this simplified prescription because whether subject-wise CV is applicable or not depends upon the dependency and distributional structure of the data, and this may or may not coincide with the intended ‘use-case.’ Finally, to avoid any doubt, I should clarify that I do not of course disagree with Saeb et al. [1] on the need to identify such obvious confounds with complex predictors, but I do not believe they are necessarily caused by train/test set dependence and thereby fixed just by using a different CV method.

Max A. Little

## Perspective by G. Varoquaux: cross-validation is important and tricky

Saeb et al. [1] discuss an important point that is well summarized by their title: the need to approximate the use-case when evaluating machine learning methods for clinical applications. The central aspect of the problem is cross-validation: How should it be done? How should the results be interpreted?

The reviewers of this important paper worried that readers might retain overly simple messages, maybe originating from the paper’s efforts at being didactic. Focused on diagnostic applications, the paper stresses the importance of subject-wise cross-validation, often performed by leave-one-subject. But there is no one-size-fits-all solution to methodological mistakes. In addition, the usage of leave-one-out cross-validation, frequent in medical informatics communities, is fragile to confounds. Here, I give my take on some issues raised by the original paper [1] and by Dr. Little’s comments.

### Confounded predictions are indeed an important problem

Given data and a target, a good predictor predicts; in other words, it tries to find statistical associations between the data and the target. Machine learning techniques can, and will, draw their predictions from effects of non-interest - confounds - or from stratifications in the data. Given two visits of the same patient with a chronic disease, who would not be tempted to conclude on a diagnostic upon recognizing the patient? If a diagnostic method is meant to be applied to new patients, it must be evaluated as such. Hence, the cross-validation strategy must test generalization to new subjects, as recommended by Saeb et al. [1].

However, if the method is meant for prognosis from continuous measurements, it may fine-tune to a subject’s data. In such a case, the cross-validation must be made by leaving out future measurements, and not full subjects. The central aspect to choose how to separate train and test data is the dependence structure between these. While most mathematical studies of cross-validation are for i.i.d. data, applications often have dependencies across observations that are due to unobserved confounding effects: samples belong to multiple subjects, and movements differ across populations. Whether these effects are confounds or not depends on the scientific or clinical question.

I have recently encountered two interesting situations that warranted specific cross-validation settings. In Abraham et al. [9], we were interested in diagnostics from imaging data of multiple sites. An important question was whether the predictive biomarkers would carry over across sites. To test this question, we measured prediction by leaving out full sites in the cross-validation. In a different application, Liem et al. [10] showed prediction of brain age from magnetic resonance images. However, it is known that elderly people tend to move more in the scanners, and that this movement has a systematic effect on the images. To demonstrate that the prediction of brain age was not driven by movement, they showed prediction on a subset of the data specifically crafted so that age and movement were uncorrelated.

In the first section of this review, Dr. Little correctly points out that, in the presence of a confounds changing the cross-validation method can measure the impact of a confound but not fix it. More so, it may lead the predictors to underfit, in other words, to use only a fraction of the information available to predict. For instance, to demonstrate prediction of brain age independent of movement, training predictive models on a data set crafted to only have subjects with a given amount of movement would give less powerful predictors. Indeed, the required data culling would deplete the train set. In addition, it would lead to predictors easily fooled by movement: they would be applicable only on data with the same amount of movement.

### Avoid leave-one-out: cross-validation with small test sets is fragile

Beyond the dependency structure of the data, another important aspect of choosing a cross-validation strategy is to have large test sets. Unfortunately, in communities such as medical imaging, leave-one-out is the norm. In such a situation, there is only one sample in the test set, which leads to a subpar measure of the error. An erroneous intuition is that this strategy creates many folds that will compensate. The standard recommendation in machine learning is to use test sets of 10% to 20% of the data (see [11,12] for historical references and [13, 7.10] for a modern text book). Experiments on brain imaging confirm this recommendation [14].

Intuitions on cross-validation are challenging as there are multiple sources of randomness: the train data which estimate the expectancy on the family of models learned, and the test data, which estimate the expectancy on the data on which the models will be applied. However, the example of creating a balanced test set in age prediction [10] outlines the importance of having many observations in the test set. It is impossible to accumulate rich statistics on errors in small test sets. It is not legit to accumulate statistics in the union of all test sets across cross-validation folds. Indeed this would break their independence with the train sets and open the door to leaking of information from the train sets to the corresponding statistics [5].

Leave-one-out must be avoided. Cross-validation strategies with large test sets - typically 10% of the data - can be more robust to confounding effects. Keeping the number of folds large is still possible with strategies known as repeated test-train split, shuffle-split, repeated K-Fold, or Monte-Carlo Cross-Validation [2,14].

### Modeling cross-validation in heterogeneous data

Discussions are made more complex by the difficult question of what exactly a cross-validation strategy is measuring. In particular, Dr. Little mentions in the first section that models may underfit or lead to large variations when predicting across subjects. These considerations have some truth. Yet, in a clear application setting, as for a clinical purpose, the intended usage should dictate the cross-validation setting. For research purposes, understanding what drives cross-validation results is important. This may be difficult, as stressed by Dr. Little. To complement these arguments with a different perspective, I expose below conceptual tools that I find useful in thinking about this rich set of questions, though I do not claim to answer them. Intuitions can enable easy communication, but here I need to rely on mathematical formalism.

First, let us give a formalism for confounding structures: for *N* observations, we consider a variable $\mathbf {y} \in \mathbb {R}^n$ that we are trying to predict from data $\mathbf {X}\in \mathbb {R}^{n \times p}$ in the presence of confounds $\mathbf {Z}\in \mathbb {R}^{n \times k}$. The confounds could be labels denoting subject membership in the across-subject prediction case of Saeb et al. [1] or indicators of movement in Liem et al. [10]. **y** is then a function of **X** and **Z**:

(1) \begin{eqnarray*} \mathbf {y} = f(\mathbf {X} \mathbf {Z}) + \mathbf {e}, \end{eqnarray*}

(2) \begin{eqnarray*} {\rm for\, linear\, associations:}\quad \mathbf {y} = \mathbf {X} \,\mathbf {w} + \mathbf {Z} \,\mathbf {u} + \mathbf {e}, \qquad \end{eqnarray*}

where **e** is observation noise. In such model, **e** may be i.i.d. even though the relationship between **y** and **X** is not i.i.d, e.g., changing from subject to subject as in Saeb et al. [1]. This formalism is a classic way of modeling confounding effects used, e.g., in statistical testing for linear models [15].

The machine learning problem is to estimate from train data {train} = (**y**_train_**X**_train_) a function $\hat{f}_{\lbrace {\rm train}\rbrace }$ that predicts best **y** from **X**. In other words, we want to minimize an error $\mathcal {E}(\mathbf {y}, \hat{f}(\mathbf {X}))$. The purpose of cross-validation is to estimate this error. The challenge is that we are interested in the error on new, unknown, data, i.e. the expectancy of the error for (**y, X**) drawn from their unknown distribution: $\mathbb {E}_{(\mathbf {y}, \mathbf {X})}[\mathcal {E}(\mathbf {y}, \hat{f}(\mathbf {X}))]$ [2]. This is why evaluation procedures must test predictions of the model on left-out data that should be independent from the data used to train the model. Another aspect is that we would like the measure to be also an expectancy on this train data: given future data to train a machine learning method on a clinical problem what is the error that I can expect on new data? This is a reason why cross-validation procedures vary the train set by repeating the train-test split many times. They strive to measure the following quantity:

(3) \begin{eqnarray*} \mathbb {E}_{\lbrace {\rm train}\rbrace }\!\left[ \mathbb {E}_{(\mathbf {y}, \mathbf {X})}\!\left[\mathcal {E}(\mathbf {y}, \hat{f}_{\lbrace {\rm train}\rbrace }(\mathbf {X}))\right] \right]. \end{eqnarray*}

Given models (1) and (2) thatinclude a confound **Z**, (3), which gives the cross-validation error, must be refined to include **Z** in the expectancies, marginally or conditionally on **Z**. If **Z** models subjects and the goal is to predict across to new subjects, the expectancies must be marginal with respect to **Z**. This tells us that all the data from a given subject should be either in the train or the test. If the prediction is a prognosis knowing the subject’s past, the expectancies are then conditional to **Z**, and a subject should be spread between the train and test sets.

Gaël Varoquaux

## The authors’ perspective

We do not live in a perfect world, and we thus are always facing trade-offs when analyzing the performance of machine learning algorithms. All cross-validation (CV) methods, at best, estimate the true prediction error. Acquiring the true prediction error would require infinite amounts of usage case data. In this sense, we completely agree with Dr. Varoquaux on the complexity of the process, which is partially reflected in the review by Dr. Little as well. We have also encountered similar complex scenarios in our own work, where subject-specific models needed to be cross-validated across time, e.g., in cases where tracking, not diagnosis, was the objective [16,17]. We thank both Dr. Varoquaux and Dr. Little for bringing these additional discussions and didactics to this important problem. However, in his review, Dr. Little has criticized three of our claims that (i) subject-wise CV mimics the use-case, (ii) subject-wise CV will, under the right assumptions, estimate the out-of-sample prediction error, and (iii) record-wise CV underestimates use-case prediction errors. We will critically review these three points here.

### When subject-wise CV mimics the use-case scenario

If we are to use machine learning to diagnose a disease, we want to generalize from known subjects, some having the disease and some not, to new patients who have to be diagnosed in the future. In this setting, subject-wise CV obviously mimics the use-case scenario by dividing the data set into known (train) and future (test) subjects. Therefore, we are not clear why Dr. Little asks if “subject-wise can be a replacement for record-wise.” For such a wide set of use-case scenarios, that is not even a question. Record-wise CV would, on a reasonably small data set, readily allow diagnosing a disease using any feature that can identify the individual. For example, we would be able to diagnose Parkinson’s disease from subjects’ nose length.

Now, there are also other scenarios: we may want to ask if a patient develops a disease, based on past baseline measures of the same patient and new data. In this case, using data from the same patient is mandatory. Here, our machine learning system needs to use information from the same subject to predict their future state. However, record-wise CV, which randomly splits data into train and test sets regardless of time, is still not mimicking the use-case scenario. Using record-wise CV here would mean detecting the disease based on future knowledge about the disease state of a subject.

### When subject-wise CV correctly estimates the out-of-sample prediction error

When we build a machine learning system that diagnoses a disease, then we must consider how often it will misdiagnose the disease on new subjects. Provided that subjects are recruited randomly from the target population, a subject that has not been seen by the algorithm is, for all practical purposes, indistinguishable from other people in the population who are not in the study. However, there are situations where this assumption will not hold: if our data set is small, even if the algorithm has not seen those other subjects, the algorithm developer certainly has, and may therefore bias the meta-parameters of the algorithm. As such, the algorithm will implicitly contain information from all data points, which means that whenever we try something on our test set, we are using the test set information. Therefore, the most important issue with subject-wise CV is the implicit re-use of test data. To deal with this problem, we can use large data sets where train and test sets are completely isolated from the beginning or run a pre-registered replication study. In this sense, we agree with Dr. Little that even subject-wise CV is not sufficiently conservative. However, subject-wise CV will still be a much more meaningful metric than record-wise.

### When record-wise CV underestimates the use-case prediction error

Quite simply, whenever there is any subject-specific component in the data, there will likely be a bias. In fact, Fig. 3 of our paper shows this dependency in simulation. The idea is also intuitive: in a small cohort, we would be able to use machine learning to “diagnose” any disease based on any subject-specific feature (e.g., the nose length), because the algorithm could, at least partially, identify subjects based on those features. This would give us an obviously misguided belief that our machine learning system will work perfectly on a new cohort. For simple confounds, such as linear ones, we can use a formalism similar to that of Dr. Varoquaux and mathematically derive the biases. Obviously, we can construct cases where there is no bias despite subject-specific variation, e.g., when the subject-specific variance is impossible for the machine learning system to learn or represent. In years of experience working with biomedical data, we have yet to see signs of such variance. In other words, the biases are mathematically predicted, are intuitively obvious, and are experimentally demonstrated by our paper.

In summary, we agree with Dr. Little and Dr. Varoquaux on the complexity of the cross-validation problem and are thankful for bringing this up. We can not, however, agree with Dr. Little’s three criticisms of our paper. We strongly urge scientists and clinicians who want to diagnose diseases to avoid record-wise cross-validation. Or if they do, we would like to be given the opportunity to short the stocks of the resulting start-ups.

Sohrab Saeb, Luca Lonini, Arun Jayaraman, David C. Mohr, and Konrad P. Kording

### 

Conflicts of interest

The authors declare no competing interests.

## Supplementary Material

GIGA-D-17-00022_Original_Submission.pdf

Supplement Files
